# Effects of elevated temperatures on the mechanical properties of laterized concrete

**DOI:** 10.1038/s41598-023-45591-5

**Published:** 2023-10-26

**Authors:** Joseph O. Ukpata, Desmond E. Ewa, Joseph U. Liwhuliwhe, George Uwadiegwu Alaneme, Koyonor E. Obeten

**Affiliations:** 1 Department of Civil Engineering, University of Cross River State, Calabar, Nigeria; 2 Department of Civil Engineering, Kampala International University, Kampala, Uganda; 3 Department of Pure and Applied Chemistry, University of Calabar, Calabar, Nigeria

**Keywords:** Structural materials, Engineering

## Abstract

This study explored the impact of elevated temperatures on the residual structural properties of concrete made with a non-conventional fine aggregate such as laterite and quarry dust. In regions prone to high temperatures, such as tropical climates, the structural integrity of concrete can be compromised when exposed to elevated temperatures. Concrete samples were subjected to high temperatures (250 °C) and compared with control samples tested under normal conditions. In this research, the concrete mix was altered by replacing fine aggregates with different combinations of laterite (Lat) and quarry dust (QD) at varying percentages: 10%Lat:90%QD, 25%Lat:75%QD, 90%Lat:10%QD, 75%Lat:25%QD, and 50%Lat:50%QD. The physical properties of the constituent aggregates, including sand, laterite, quarry dust, and granite, were assessed, and an experimental mix was designed. The concrete samples underwent curing for 3, 7, 14, and 28 days, and their mechanical properties, specifically compression and flexural strength, were analyzed. The results demonstrated that as the percentage of laterite in the concrete matrix increased, there was a linear improvement in performance in terms of density, sorptivity, and strength gain. The maximum compressive strength reached 32.80 N/mm^2^ at 90% laterite replacement. However, flexural strength showed a different response, with the highest strength of 5.99 N/mm^2^ observed at 50% laterite replacement, after which strength declined with further increases in the laterite ratio. For economic and engineering considerations, it is recommended to use 25% laterite replacement with sand to produce grade 30 concrete, while 50% laterite replacement is suitable for grade-25 concrete. Importantly, the study found that a temperature of 250 °C did not significantly affect concrete strength, with changes of no more than 5%, which is consistent with expectations for conventional concrete. Furthermore, this research suggests that an optimal laterite replacement range of 25–50% should be considered when using laterite in concrete production.

## Introduction

Concrete serves as a fundamental building material renowned for its adaptability, longevity, and cost efficiency, making it indispensable in various construction applications. Nonetheless, in real-world situations, concrete structures encounter diverse environmental conditions, including exposure to elevated temperatures, which can arise from fires or high-temperature industrial processes^1^. Such exposure to heightened temperatures can have a profound impact on concrete's structural characteristics, resulting in diminished strength, rigidity, and resilience. Often, infrastructures confront elevated temperatures due to fire incidents and other factors, underscoring the importance of considering materials' resistance to high temperatures when designing and implementing such structures^2^.

Moreover, with the growing utilization of unconventional materials in construction, there is an increasing need for comprehensive studies to gain deeper insights into how these construction materials perform under varying conditions. Numerous prior investigations have sought to comprehend the behavior of diverse non-traditional concrete materials, including laterites^3,4^, palm kernel shells^5^, crushed recycled concrete^6,7^, and others. These non-conventional aggregates offer environmental advantages like reduced reliance on natural resources and waste reduction. Nevertheless, their performance under elevated temperature conditions remains a relatively unexplored area of research^8^.

Over time, there has been a notable surge in interest regarding the exploration of alternative materials to replace traditional concrete aggregates. Shanmugavel et al*.*^9^, described non-conventional concrete also known as concrete aggregate substitutes as the adding or replacement of some waste materials or fibers in the normal concrete. Also, Limbachiya et al*.*^10^ discovered that the compressive strength and modulus of elasticity of concrete made with recycled aggregate is at least two-third that of natural aggregate concrete. Baalbaki et al.^11^ proved that the volume fraction of aggregates and elastic properties are affected by elastic modulus of concrete*.* The palm kernel shell which has proven to have properties of a good concrete aggregate according to Abdul and Ganapathy^12^, is hard and good enough but is not recommendable because of its unavailability, the demand for sand fit for construction works is on the increase daily^13^.

Moreover, it has been observed that the global extraction of billions of tons of sand and gravel annually to meet the demands of the construction industry is causing significant environmental repercussions. This practice leads to the depletion of beaches and riverbeds, causing damage to public assets and exerting additional stress on industrial, commercial, and residential structures^14^. Consequently, there is a pressing need to explore alternatives to traditional fine aggregates, such as lateritic sand, to mitigate the environmental impact of sand extraction and reduce the vulnerability of coastal communities to storm damage. This can be achieved by partially replacing sand with laterite^15^.

Furthermore, the impact of elevated temperatures on concrete is influenced by various factors, including the properties of the aggregates used in the mix. Non-conventional aggregate-based concrete may exhibit different thermal responses compared to conventional concrete due to variations in mineral composition, porosity, and microstructure^16^. Therefore, gaining a comprehensive understanding of how concrete made with non-conventional aggregates behaves under elevated temperature conditions is essential for ensuring the structural integrity and fire resistance of these environmentally sustainable concrete blends. Research conducted by Xie et al.^17^ evaluated the thermal properties of laterite concrete by conducting thermal conductivity tests. The results indicated that the thermal conductivity of laterite concrete decreased with increasing aggregate size and water-cement ratio.

Also, the research by Nguyen et al.^18^ investigated the compressive strength of laterite concrete specimens subjected to high temperatures. The study revealed a reduction in compressive strength with increasing temperature, highlighting the need to consider the thermal response of laterite concrete in structural design. A study conducted by Wang et al.^19^ used SEM to analyze the microstructural changes in laterite concrete after exposure to high temperatures. The results showed the formation of micro cracks and the decomposition of hydration products, indicating the deterioration of the concrete's mechanical properties. Additionally, research by Liu et al.^20^ used FEA to analyze the temperature field and stress distribution in laterite concrete elements subjected to fire. The modeling results provided insights into the thermal response and structural performance of laterite concrete structures. Based on the literature review of previous research in the study area, the following observed research gaps were identified;

i. Existing research lacks a comprehensive understanding of how laterized concrete incorporating non-conventional materials reacts to high temperatures.
ii. There is limited research examining the thermal stability of laterized concrete in comparison to traditional concrete.
iii. The construction sector lacks clear guidelines for the utilization of non-traditional aggregates such as laterite in concrete mixtures.

This research endeavor aims to bridge these gaps in existing knowledge by exploring the response of laterized concrete to elevated temperatures. It will offer valuable insights and contribute to the establishment of comprehensive guidelines for sustainable construction practices.

The primary objective of this research is to investigate how elevated temperatures impact the elastic structural characteristics of concrete that incorporates non-traditional aggregates such as laterite and quarry dust. Additionally, it seeks to assess the mechanical and morphological properties of concrete samples subjected to controlled heating within a high-temperature chamber. The experimental plan will involve the substitution of fine aggregates with various percentages of laterites and quarry dust, ranging from 10 to 90%, to gauge their influence on the concrete's response to elevated temperatures. This study will contribute valuable insights into the fire resistance and thermal stability of sustainable concrete formulations. The outcomes of this research will offer a deeper understanding of how elevated temperatures affect the mechanical attributes of these concrete compositions. This knowledge will empower engineers and designers to make well-informed decisions regarding the utilization of non-traditional aggregates in construction projects located in fire-prone environments. Ultimately, the study's overarching goal is to enhance the resilience and safety of concrete structures exposed to high-temperature conditions, while promoting sustainable construction practices.

## The effect of fire on conventional concrete

Concrete, though not a refractory material is combustible and has good fire-resistant properties. The ability of the concrete to sustain the effects of heat and the resulting reaction of water without excessively weakening, cracking, or spalling; the concrete's thermal conductivity; and the concrete's coefficient of thermal expansion are the main variables that influence a structure's ability to resist fire. In the case of reinforced concrete, fire resistance depends not only on the kind of concrete but also on the thickness of the cover over the reinforcement. The fire produces huge temperature gradients, and as a consequence, the top layers resist spalling and sputtering off the colder interior. The expansion of the reinforcement bars horizontally and vertically is made worse by heating, which causes the reinforcement to lose its connection and become weaker^21,22^.

When heated, the aggregates in mortar and concrete expand gradually, whereas the hydrated products in cement once it has expanded to its greatest extent shrinks. The concrete weakens and cracks as a result of these two antagonistic activities. In terms of how they behave when heated, the different aggregates employed differ greatly. Quartz, the main mineral found in sand, gravel, granites, and gravels, grows gradually up to a temperature of around 573 °C. It suddenly expands by 0.85% when this temperature is reached. A descriptive effect of this expansion is seen in concrete's stability. If quartz is the most common mineral in the aggregate, the fire resistance of concrete is at its lowest^23^.

Fire has significant effects on concrete structures, leading to changes in their mechanical, thermal, and chemical properties. To mitigate the effects of fire on concrete, various strategies can be employed, such as the use of fire-resistant additives, fireproofing coatings, and appropriate structural design considerations^24^. Additionally, fire protection measures, such as the installation of fire-resistant barriers and early detection and suppression systems, are essential for minimizing the impact of fire on concrete structures. Overall, understanding the effects of fire on concrete is vital for designing fire-resistant structures, implementing appropriate safety measures, and ensuring the resilience and structural integrity of concrete buildings and infrastructure in fire situations^25^. Some of the primary effects of fire on concrete include:

i. Loss of strength: Elevated temperatures during a fire can cause a reduction in the compressive strength of concrete. This is primarily due to the dehydration of cement paste and the decomposition of calcium hydroxide, which weakens the concrete matrix. The severity of strength loss depends on factors such as the duration and intensity of the fire^26^.
ii. Cracking and spalling: Concrete subjected to high temperatures may undergo cracking and spalling. The heat causes moisture trapped within the concrete to turn into steam, resulting in an increase in internal pressure. The buildup of pressure can lead to explosive spalling, where fragments of concrete break off from the surface. Cracking and spalling compromise the structural integrity of the concrete^27^.
iii. Thermal expansion and stress: Fire causes concrete to expand due to thermal effects. The differential expansion of the concrete components, such as the aggregate and cement paste, can induce internal stresses. These stresses may lead to cracking and distortions in the concrete, further weakening its structural performance^2^.
iv. Loss of durability: The exposure to high temperatures can adversely affect the durability of concrete. The heat can accelerate chemical reactions, such as carbonation and alkali-silica reaction, which can lead to long-term deterioration and reduced service life of the structure^28^.
v. Changes in microstructure: Fire can cause changes in the microstructure of concrete, including the decomposition of hydrated cement compounds and the formation of new phases. These alterations can affect the pore structure, density, and overall mechanical properties of the concrete^29^.

### Effects of aggregate types on thermal properties of concrete

The type of aggregate used in concrete can have significant effects on its thermal properties. The thermal properties of concrete, such as thermal conductivity, thermal diffusivity, and specific heat capacity, play a crucial role in determining how concrete responds to changes in temperature^30^. The variation in thermal properties of concrete due to different aggregate types can impact the overall thermal behavior of structures. It can affect the rate of temperature rise or drop within the concrete, as well as the distribution of temperature throughout the structure. This, in turn, can influence factors such as thermal stress development, potential for cracking, and the overall thermal performance of the concrete^31^. It is important for engineers and designers to consider the thermal properties of concrete and select suitable aggregate types based on the specific requirements of the project. By understanding the effects of aggregate types on the thermal properties of concrete, it becomes possible to optimize the material composition for better thermal performance, energy efficiency, and durability of concrete structures in various temperature conditions^17^. Howlader et al*.*^32^ determined the thermal properties of concrete manufactured with different categories of aggregates. The research focus on the measurement of thermal conductivity of conventional aggregate and brick aggregates respectively. It was concluded that the specific heat of concrete made with bricks is 13% greater than the concrete having stone chips. The research also concluded that thermal conductivity has a linear relationship with thermal diffusivity in both types of aggregates. The duration of fire and the maximum temperature reached vary over a wide range. Temperature of 1000 to 1100 °C in fires lasting from 1 to 2 h have been observed more frequently than 1300 °C.

An accurate estimation of the performance characteristics of structures damaged in a fire helps in taking effective measures of- restoration. The performance characteristics take into account the physio-chemical and mechanical properties of the materials burnt and that of heated concrete. There is an accumulation of irreversible damages of mechanical and physio-chemical factors. Under mechanical factors, creep, cracking, shrinkage and plastic deformations may be classified, while under physio-chemical factors, corrosion, absorption and degradation etc. may be classified^33^. Here are some of the effects of aggregate types on the thermal properties of concrete:

i. Thermal conductivity: The choice of aggregate can influence the thermal conductivity of concrete. Different types of aggregates have varying thermal conductivities, which is a measure of how well heat can transfer through the material. Aggregates with higher thermal conductivity, such as dense and heavy aggregates like granite or basalt, can result in concrete with higher overall thermal conductivity^34^.
ii. Thermal diffusivity: Aggregate types also affect the thermal diffusivity of concrete. Thermal diffusivity represents the speed at which heat can travel through the material. Aggregates with higher thermal diffusivity, such as aggregates with good thermal conductivity and low density, can lead to concrete with faster heat transfer properties^35^.
iii. Specific heat capacity: The specific heat capacity of concrete is influenced by the type of aggregate used. Specific heat capacity refers to the amount of heat energy required to raise the temperature of a material by a certain amount. Aggregates with higher specific heat capacity, such as aggregates with high density, can contribute to concrete with a higher capacity to absorb and store heat^36^.

### Effect of high temperature on cement paste

It is accepted that in the temperature range of 4 to 80 °C the hydration process of ordinary Portland cement retain chemically stable, except possibly for the slight increase in the C/S ratio, where C and S represent C90 and ST02 respectively^37^. Neville^38^ stated that concrete loses it absorbed water in the range of 65 to 80 °C and its interlayer water in the 80 to 100 °C range. The increase in temperature up to 100 °C may supply the un-hydrated cement particle, with needed activation energy to enforce its hydration. This could be beneficial to concrete strength development if the W/C ratio of the gel is correct.

When temperatures are raised from 100 to 200 °C, the material properties of the cement past raised begins to lose its stability due to a weak physical chemical reaction. In this temperature range, the evaporable moisture plays a dominant role in reducing cohesive forces between C-S-H layers and their gel surface energy. And thus the cement gel shrinks and the strength reduces^39^. In this temperature range, dissociation of calcium hydroxides does not occur generally. Actually, the decomposition of calcium hydroxide into lime and water vapour during heating is not critical in terms of strength loss, but it leads to serious damage due to lime expansion during the cooling period. This indicate the harmful effect of cooling the heated concrete by gradual cooling in natural air is also harmful due to the absorption of heated concrete containing lime (CaO) in its cement gel logically, it is concluded that cooling concrete by water (which is considered gradual cooling) has every harmful effect in reducing concrete compressive strength when compared to the effect of cooling in the natural air (which considered gradual cooling)^40^. In higher temperature ranges 400–600 °C, a series of reaction in hardened cement paste may activate. This reaction starts with the completion of desiccation of the poor water system, followed by (H) and the decomposition of hydration products. Decomposition of C-S-H or C-S-H (H) generally occurs at 600–700 °C. the breakdown of C-S-H may take place at 800 °C and results in slight increase in the residual strength. The chemical decomposition and loss of the chemically bonded water set the stage for melting at temperature above 900 °C^41^.

High temperatures can have significant effects on cement paste, which is the binding material in concrete. Understanding the effects of high temperature on cement paste is crucial for assessing the performance and durability of concrete structures exposed to fire or other elevated temperature environments^42^. It helps engineers and designers make informed decisions regarding material selection, structural design, and fire-resistant measures to ensure the safety and integrity of concrete structures in such conditions. Additionally, it guides the development of high-temperature resistant cementitious materials and protective measures to mitigate the detrimental effects of high temperatures on cement paste and overall concrete performance^43^. When cement paste is exposed to elevated temperatures, several changes occur, including:

i. Dehydration: One of the primary effects of high temperature on cement paste is dehydration. The heat causes the evaporation of water from the cement paste, leading to the loss of moisture. Dehydration can result in a decrease in the volume of the paste and the formation of microcracks, which can weaken the material^44^.
ii. Calcium hydroxide decomposition: Cement paste contains calcium hydroxide (portlandite), which can decompose at high temperatures. The decomposition of calcium hydroxide releases water vapor and leads to a reduction in the pH of the paste. This can affect the chemical stability of the paste and potentially contribute to the deterioration of concrete^45^.
iii. Strength reduction: Elevated temperatures can cause a reduction in the strength of cement paste. Dehydration and the decomposition of calcium hydroxide can weaken the bonding within the paste, leading to a decrease in compressive strength and other mechanical properties. The severity of strength reduction depends on the temperature and duration of exposure^38^.
iv. Microstructural changes: High temperatures can induce significant microstructural changes in cement paste. It can cause the formation of new phases, such as calcium aluminate hydrates and anhydrous phases, which can alter the paste's structure and properties. These changes can impact the paste's density, porosity, and overall durability^46^.
v. Cracking: Thermal expansion mismatch between the cement paste and other components, such as aggregates or reinforcement, can lead to the development of cracks in the paste. The differential expansion and contraction can generate internal stresses, resulting in cracking and potential loss of integrity^39^.

### Lateritic soil

Lateritic soil, also known as laterite, is a type of soil that is commonly found in tropical and subtropical regions. It is formed through weathering processes that occur in hot and humid climates, where intense rainfall and high temperatures promote the decomposition of rocks and minerals^47^. The importance of lateritic soil in engineering field cannot be over emphasized, Field observation show that the predominant content of the admixture will mainly be sand. However, the sand occurs with different amounts of clay, silt and grave. The sand is expected to influence the engineering behavior of the cohesive soil. Knowledge of the influence of sand on strength and compressibility parameters of the soils is fundamental in the interpretation of their properties for engineering design^48^.

Hence, Laterite can simply be defined chemically as a material having the ratios of silica to sesquioxide represented by molecular silica-alumina ratio: SiO_2_/Fe_2_O_3_, in the range of 1.33–2.0. Laterite soil with ratios less than 1.33 are considered indicative of true laterites, while those between 1.33 and 2 indicate laterite soils and those greater than 2 are non-laterite tropically weathered soils. Laterized concrete is defined as the replacement of fine aggregate (sand) with laterite at different proportions in the component of concrete^49^. The quest of having concrete at a cheaper rate and at varying temperatures has prompted many researchers to work on laterized concrete. Nkanang et al.^50^ conducted a study on laterized concrete and found that as the percentage of laterite in the mix increased, the compressive strength decreased. They also observed a decrease in flexural strength with higher laterite content. Also, Obilade et al.^51^ investigated the effect of different laterite particle sizes on the compressive strength of laterized concrete. They reported that smaller particle sizes of laterite resulted in higher compressive strength compared to larger particle sizes.

## Methodology

Ordinary Portland Cement (OPC), lateritic sand, river sand and quarry dust were used for all mixes in this research. In obtaining the effect of elevated temperatures, the study considered 0 °C and 250 °C. The concrete strength was studied for 3, 7, 14, and 28 days respectively, the experimental program and concrete mix design for this research study is presented in Tables 1, 2.

**Table 1:** Experimental program.

Material characterization	References		
Sieve analysis	BS EN 933-1:2012		
Specific gravity	BS EN 1097-6:2013		
Aggregate impact	BS EN 1097-2:2010		
Oxide composition	BS 12:1996		

Testing of hardened concrete samples	Sample cube	Test curing age	Standard specifications
Compressive strength	100 × 100 × 100	3, 7, 14, 28	BS EN 12390-3:2019
Flexural strength	400 × 100 × 100	28	BS EN 12390-5:2019
Water absorption	200 × 100	28	BS EN 1097-6:2013

**Table 2:** Concrete mix design.

Code	Mixture aggregates combinations	Cement (kg/m^3^)	Water (kg/m^3^)	Sand (kg/m^3^)	Laterite (kg/m^3^)	Quarry (kg/m^3^)	Course aggregate (granite chippings)	W/C
A1	(Control)	333	210	672	–	–	1195	0.63
A2	10%L: 90%QD	328	210	–	76.08	684.72	1141.2	0.64
A3	25%L:75%QD	328	210	–	190.2	570.6	1141.2	0.64
A4	90%L:10%QD	328	210	–	684.72	76.08	1141.2	0.64
A5	25%QD:75%L	328	210	–	570.6	190.2	1141.2	0.64
A6	50%QD:50%L	328	210	–	380.4	380.4	1141.2	0.64

L: Laterite, QD: Quarry dust.

### Materials

#### Cement

The Lafarge cement brand of ordinary Portland cement was used for this purpose. This cement sets and hardens when it interacts with water chemically. I ensured that it is kept in a damp-free environment to avoid lump formation. Cement was gotten from Lafarge cement plant at Mfamosing, this is the Ordinary Portland Cement (OPC) which undergoes hydration when it comes in contact with water and conforms to BS EN 197-1:2011. The cement was kept away from wet and extreme cold weather to avoid it from forming lump.

#### Water

Water is what aids the binding of cement; hence, it is importance in this research. It brings about the hydration process of cement and aids the overall reaction between cement and other materials in it. Domestic Portable tap water inside the lab was used throughout the laboratory aspect of this research work and it met the ASTM C1602-12 water specification for concrete works.

#### Aggregate

Aggregates as an important constituent in concrete contributes largely to its economy and also promotes the body building of concrete, while minimizing its shrinkage effect. In this research two different fine aggregates namely laterite and quarry dust were used, while the conventional fine sand obtained in from river bed in Akwa Ibom State, Nigeria with particle size of 6 mm maximum, and 2.4 fineness modulus was used as control.

##### Laterite

**Figure 1:**
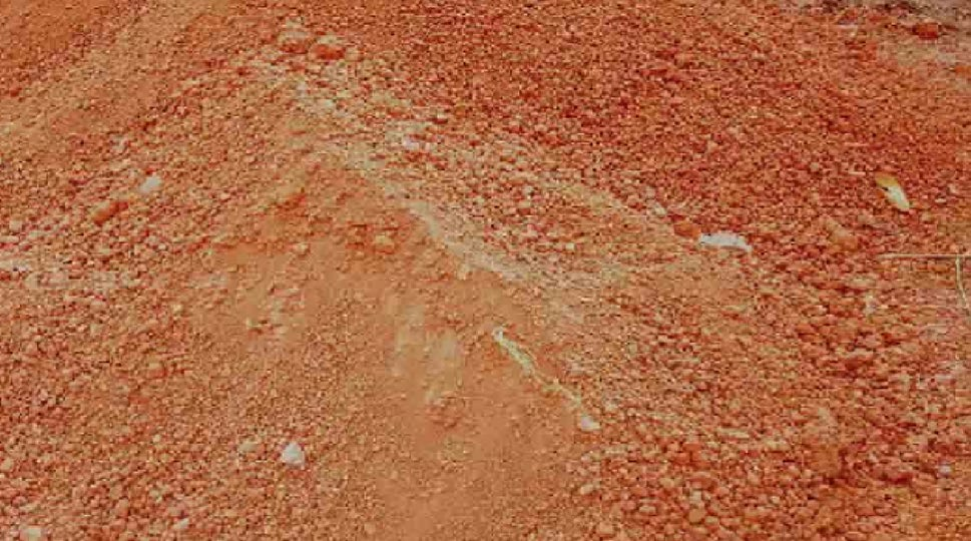
Lateritic soil for the experiment.

Lateritic sand as one of the fine aggregates used in this work was gotten from a borrowed-pit site at Goodluck Jonathan bypass, Calabar at a depth of 2.5 m. the lateritic soil samples used for the test experiment in this research study is shown in Fig. 1

.

##### Quarry dust

This material was obtained from the rich deposit at Mark Sino quarry site at Akamkpa local government area of Cross River State, and it is a local government located in the Southern part of Cross River State which is approximately 25 min drive from Calabar City.

#### Coarse aggregate

Crushed granite was the coarse aggregate used with maximum and minimum sizes of 20 mm and 5 mm respectively produced at Akamkpa quarry site conformed to BS EN12620.

### Methods

The methods involved characterization of the concrete constituent materials, preparation of concrete samples, and testing of hardened concrete samples for different structural properties and water absorption characteristics. Lateritic sand and quarry dust was used to substitute the conventional sand in different proportions from 10 to 90%. In order to develop controls for the experimental works, important related literatures were reviewed and standard/codes of practice were also applied. In the laboratory, experiment was carried out in this work by testing the fresh and hardened concrete^52^.

#### Specific gravity test

The specific gravity test is a commonly conducted laboratory test to determine the density or specific gravity of a solid material. It is often performed on construction materials such as aggregates, soils, and cementitious materials, including concrete. The specific gravity of a substance is the ratio of its density to the density of a reference material, typically water. The test helps to assess the quality, composition, and properties of materials, and it can provide valuable information for engineering and construction applications. The specific gravity of a material is a dimensionless value and is typically reported as a ratio or a decimal. A specific gravity greater than 1 indicates that the material is denser than water, while a specific gravity less than 1 indicates it is less dense^53^. The specific gravity test by the pycnometer method is a commonly used technique to determine the specific gravity of solid materials. This method involves the use of a pycnometer, which is a glass or metal container with a known volume. To calculate the specific gravity of the test materials, we deploy the formula below using the formula:

$${\text{Specific Gravity}}\, = \,\left( {{\text{Weight of Sample in Air/}}\left( {\text{Weight of Sample in Air}} - \text{Weight of Pycnometer with Water} - {\text{Weight of Empty Pycnometer}} \right)} \right) \, * \, \left( {\text{Density of Water at Test Temperature}} \right).$$

#### Aggregate impact test

The aggregate impact value (AIV) test is a standard test conducted on aggregates to determine their resistance to sudden impact or shock. It provides an indication of the aggregate's toughness and suitability for use in construction applications, particularly in road construction. A representative sample of the aggregate were obtained, usually with a minimum size of 10 mm. Ensure the sample is clean, dry, and free from any contaminants. The test samples were aggregates sized 10.0 mm to 12.5 mm. The aggregates was dried by heating at 100–110 °C in the oven for a period of 4 h and cooled. The test sample is placed in the cylindrical measure and compact it using 25 strokes of the tamping rod. Each layer should be uniformly tamped. The measure with the compacted aggregate is then placed in the impact testing machine. A total of 15 blows evenly distributed on the aggregate is applied, each blow being delivered by a standard hammer weighing between 13.5 to 14 kg. The height of fall for each blow is usually 380 mm. After the impact, remove the aggregate from the measure and sieve it through a 2.36 mm sieve. The aggregate impact value for the test samples is calculated using the formula:

$${\text{Aggregate Impact Value}}\, = \,\left( {{\text{Weight of Material Passing through 2}}.{\text{36 mm Sieve}}/{\text{Original Weight of the Test Sample}}} \right) \, *{ 1}00.$$

#### X-ray fluorescence test

X-ray fluorescence testing provides valuable information about the elemental composition of a sample, including major, minor, and trace elements. It allows for the identification and quantification of elements ranging from sodium to uranium, depending on the capabilities of the XRF instrument. It is commonly applied in various industries, including mining, geology, environmental analysis, materials science, and archaeology. XRF provides non-destructive and rapid analysis, making it a valuable tool for quality control, research, and exploration purposes. This was done by X-ray fluorescence using an Axios PANAlytical machine with System ID 206104, Type number PW4400/10, S/N DY3222 and MFG date 2015-12 to determine the oxides composition of the samples^54^.

#### Compressive strength test

**Figure 2:**
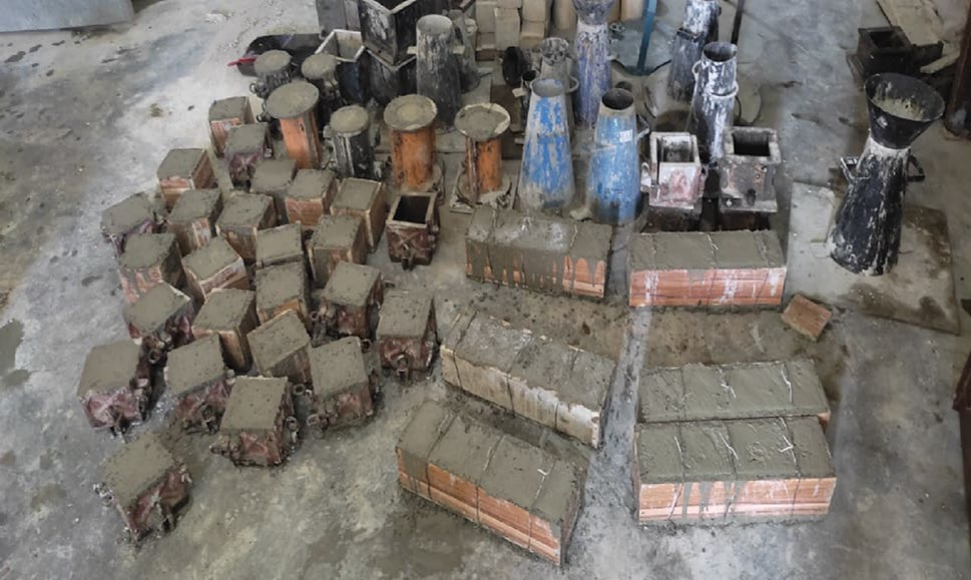
Freshly cast concrete cubes, cylinders and beams.

The compressive strength test is a standard test conducted on concrete to determine its ability to withstand compressive loads. It is one of the most important tests for evaluating the quality and durability of concrete in various construction applications. The constituent materials used for this research are cement, quarry dust, laterite, crushed granite and water. Lateritic sand and quarry dust were added to the mix to replace conventional fine sand at various percentages of 10–90%. The sample should be taken in accordance with BS EN 12390 standards. Generally, cylindrical or cube-shaped specimens are commonly used for testing. The fresh concrete were filled in the cubic molds of 150 mm × 150 mm × 150 mm. the concrete cubes were removed from the molds after 24 h and immersed into the curing tank for varying curing durations of 3, 7, 14, and 28 days respectively. Place the hydrated specimen centrally on the compression testing machine, ensuring that it sits level and properly aligned with the loading axis. Apply a gradual and continuous load at a specified rate, typically around 0.2 MPa/s, until the concrete specimen fails under compression and record the maximum crushing load applied to the specimen^55^. The freshly mixed concrete cubes, cylinders and beams is shown in Fig. 2. The compressive strength of the concrete specimen is calculated using the formula:

$${\text{Compressive Strength }} = {\text{ Maximum Load/Cross-sectional Area of the Specimen}}$$

#### Flexural strength test

**Figure 3:**
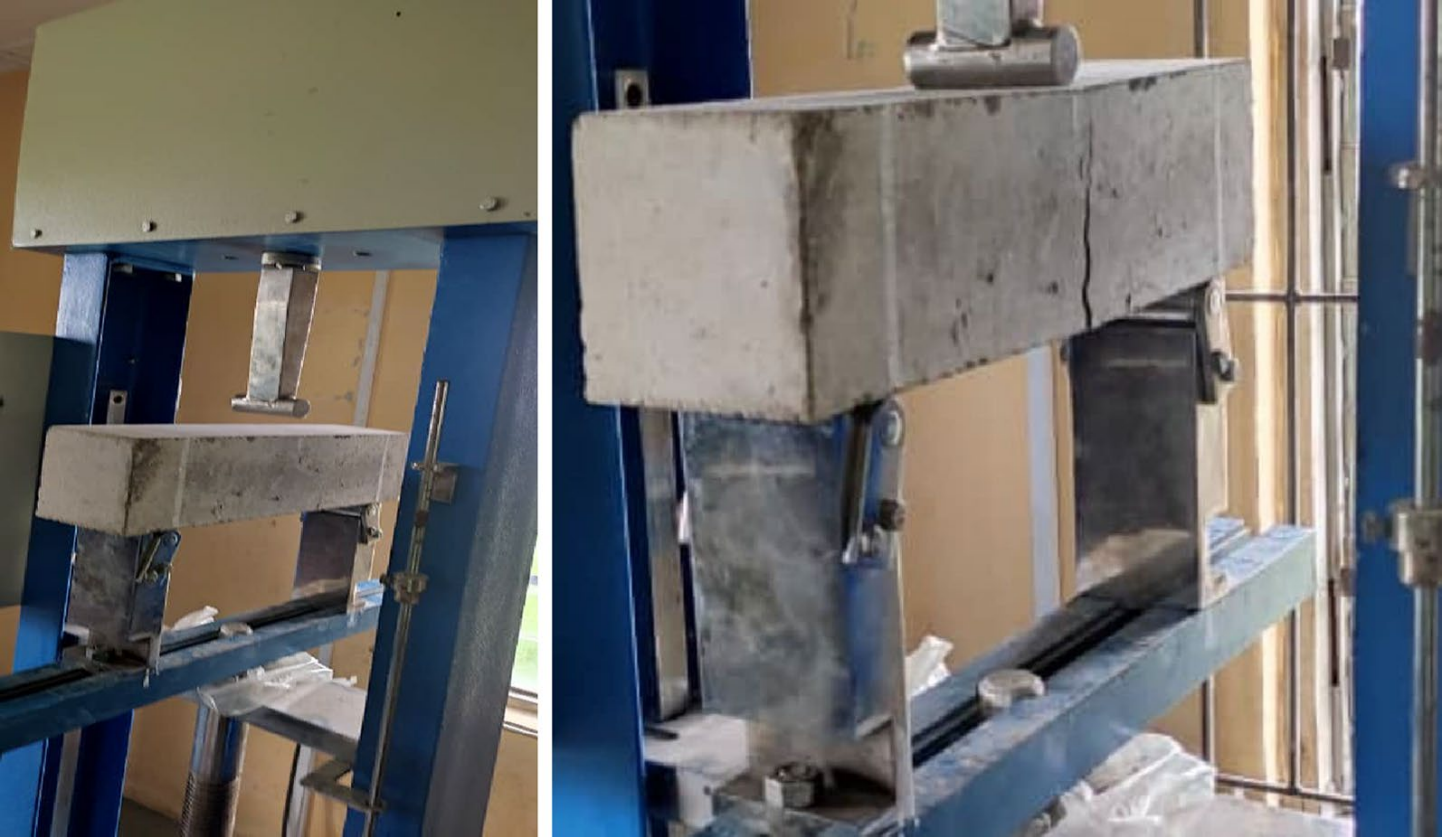
Beam sample undergoing flexural strength test.

The flexural strength test, also known as the modulus of rupture test, is conducted on concrete to determine its ability to resist bending or flexural stresses. This test provides valuable information about the concrete's strength and its ability to withstand loads that cause bending or deflection. A concrete beam of 100 mm × 100 mm × 400 mm dimension was cast as sample for flexural test. In casting this beam, a wooden mould of the size above was fabricated in the wood products engineering department of the university, this mould was properly lubricated before the freshly mixed concrete was introduced into it in 3 layers of approximately 30 mm thickness, then vibrated with a vibration table for an average of 15min until all air bobbles/pour spaces are filled. After the last layer which is approximately 40 mm tick the surface was dressed to achieve smooth finishing. The concrete beams were removed from the molded after 24 h and cured for 28 days. The beam specimen are then placed on the supports of the flexural testing machine, ensuring that it rests horizontally and aligns with the loading axis. Apply a gradual and uniform load at the midpoint of the specimen until it fractures or reaches the desired deflection, as per the testing standard BS EN 12390-5. Record the maximum applied load and the corresponding deflection^56^. The experimental setup for the flexural test is shown in Fig. 3. Calculate the flexural strength of the concrete specimen using the formula:

$${\text{Flexural}}\;{\text{Strength}} = {\text{Maximum}}\;{\text{Load}}/({\text{Width}} \times {\text{Height}}^{2} )$$

### Sorptivity test

Sorptivity is the ability of a materials to absorb water and transmit it through the pours by capillary action^57^. The tests were carried out for the sand replaced with quarry dust and laterite in the concrete mix and the control. The sorptivity test is conducted on concrete to measure its ability to absorb and transport water through capillary action. This test provides valuable information about the permeability and durability of concrete in relation to water absorption. Concrete beams of 200 × 100 × 100 mm were cast the same way they were cast for flexural strengths test with a binder to aggregate ratio of 1:2.3:3.2 and w/c ratio of 0.63. Three concrete samples were cast for each percentage replacement (10%, 25%, 50%, 75% and 90%). The concrete samples were cured for 28 days, then removed from the curing tank, allowed to drain and sun-dried in a temperature between 28 and 38 °C. The drying took up to 3 days during which the weights of the samples were checked at intervals until the difference in weight remains relatively the same. The samples were then removed from the sun and used for the test. The heated samples were places in a kiln and heated for 250 °C, allowed to cool at room temperature, then removed and used for the test^58^.

A steel mesh was placed in a stainless steel tray containing water to a level that reaches 5 mm on the rectangular beam. The initial weight of each concrete sample was taken as W_o_ (g). The concrete sample were placed vertically on the mesh with the 100 mm × 100 mm faced touching the mesh to 5 mm water level and allowed for 4 min timed with a stop watch. After 4 min, each concrete sample was removed and placed on a damp towel to mob-off the excess water on the body. The sample was immediately placed on the digital weighing balance to record the weight at four minutes, taken as W_4_ (g). The sample was placed back on the mesh and the stop watch placed to continue timing for the process was repeated for 9, 15, 25, 36, 49 and 56min respectively. The weight was recorded as W_9,_ W_15,_ W_25,_ W_36,_ W_49_ and W_64_ respectively. For each percentage replacement, the average rate of absorption in (g/mm^2^) × (10^–3^) was plotted against the square root of time (√t). The slope of the graph gave the sorptivity of the material^59^.

### Consent to participate

All authors were highly cooperative and involved in research activities and preparation of this article.

## Results and discussion

### Physical properties of materials

Concrete is composed of various ingredients that contribute to its physical properties. The physical properties of concrete ingredients can vary depending on factors such as the specific type and source of the ingredient, manufacturing processes, and regional variations. Understanding the physical properties of concrete ingredients is crucial for designing and producing concrete with desired characteristics. These properties influence factors like workability, strength, density, and durability. It is important to consider the specific requirements of each ingredient and their interactions to achieve the desired concrete properties^60^.

#### Specific gravity

The respective specific gravity of constituent aggregate materials: laterite, quarry dust and crushed rocks from the test results are 2.2, 2.6 and 2.9 respectively. The values are in agreement with the research findings of Ukpata et al*.*^3^ and Ilangovana et al*.*^61^

#### Aggregate impact value

The aggregate impact value for the coarse aggregate (crushed granite) was 6.7% thereby making it exceptionally strong, as it is less than 10%. The particle sizes of fine aggregates (sand, laterite, and quarry dust) ranged from 0.06 to 4.75 mm, while coarse aggregate (crushed granite) ranged from 5 to 20 mm in sizes. With the results above, the aggregate was generally found to be suitable for concrete work, since they meet the BS EN 1097-2:2010 specification.

#### Particles size distribution

**Figure 4:**
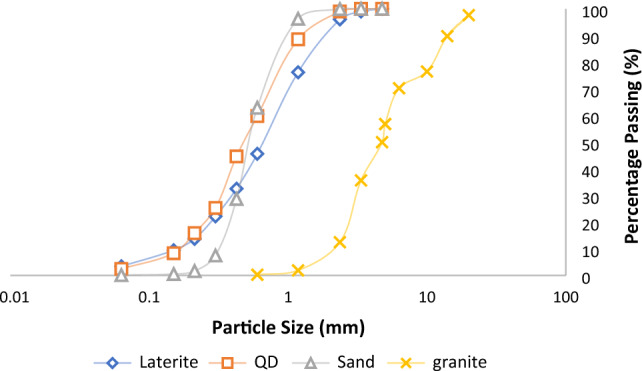
Particle size distribution for test aggregates.

The particle size distribution curve for the fine aggregate (Laterite, Quarry dust and fine river sand) and coarse aggregate (granite) is presented in Figs. 3, 4. The obtained grain size distribution data, overall, reveal well-graded sand and gravel particles that also adhere to the BS EN 933-1:2012 criteria for increased longevity of concrete capability^62^.

### Chemical properties

Concrete is composed of various materials that have distinct chemical properties. Understanding the chemical properties of concrete materials is crucial for assessing compatibility, potential reactions, and long-term performance of concrete structures. It helps in mitigating adverse effects and optimizing the composition and mix design for desired chemical behavior and performance. The oxide composition of the various materials; sharp sand, quarry dust, crushed granite, laterite and cement were obtained from the XRF test results as shown in Table 3 below.

**Table 3:** Oxides composition of materials.

Oxides	Fine sand%	Quarry dust%	Crushed granite%	Laterite%	Cement OPC 42.5N%
SiO_2_	71.77	76.59	67.52	91.03	17.61
AL_2_O_3_	2.91	6.83	6.26	3.64	4.02
Fe_2_O_3_	1.29	3.4	8.51	1.16	2.94
CaO	1.79	4.61	7.24	2.08	60.40
MgO	0.00	0.00	2.77	0.00	2.10
SO_3_	0.00	0.07	0.09	0.02	2.22
K_2_O	1.34	5.23	3.20	0.08	0.88
Na_2_O	0.00	1.34	1.11	0.00	0.03
LOI	0.70	1.85	2.8	1.95	8.39
Sum	99.8	99.92	99.5	99.96	98.59

From the results of XRF in Table 3, laterite has relatively close oxides properties to sharp sand in terms of clinker requirement; also, the content of magnesium oxide (MgO) did not exceed 5.0%. It is also discovered that laterite has very high SiO_2_ content and very low CaO content. This however satisfied the requirement of Natural Pozzolans, which is that they should have a combined sum of SiO_2_, Al_2_O_3_ and Fe_3_O_2_ of at least 70% of the total oxides^63^. Also, it can be seen that the silica (SiO_2_) content in sharp sand, quarry dust, crushed granite and laterite falls within the same neighborhood. It is also observed that silica has the highest percentage composition amongst other oxides in all the materials. Silica (SiO_2_) is important in cement as it imparts strength to the cement due to the formation of di-calcium silicate and tri-calcium silicate. However, excess silica causes slow setting hence should be checked^64^.

*Alumina (Al*_*2*_*O*_*3*_*)* On the other hand appears to be second in composition, it is responsible for the setting time of concrete; it imparts quick setting property to the cement. It is also worthy of note that, excess amount of alumina weakens the cement^65^.

*Iron oxide (Fe*_*2*_*O*_*3*_*)* The next oxide above is Iron oxide (Fe_2_O_3_), as can be seen above, the parentage composition of Iron oxide in laterite (1.16), is almost the same with that of conventional sand (1.29). The presence of iron powder in cement improves the compressive strength and porosity but decrease the slump flow. Also, the water adsorption of the concrete increases with increasing amount of Fe in it. However, high amount of iron powder in a cement sample, decreases the workability of the concrete^66^.

*Calcium oxide (CaO)* This is otherwise known as Lime. This oxide contributes to the mechanical strength of concrete, Attah et al.^67^, stated that the presence of calcium oxide in concrete gives it a high degree of mechanical strength, hydrates heat, and x-ray diffraction. The results above shows that there is a resemblance between the lime content in sand and that of laterite.

*Sulphur trioxide (SO*_*3*_*)* Some research have implied that increasing Sulphur trioxide can enhance the expansive performance of cement material and prolong the setting time of the cement but the strength of the cement will decrease if the content of the Sulphur trioxide is more than 4%; especially in Portland cement^68^.

*Sodium oxide (Na*_*2*_*O) and potassium oxide (K*_*2*_*O)* These can be called Alkalis oxide because when they react with water they form alkalis (Mohammed and Ali). The significant proportions of alkalis in cement can cause a quick setting, reduce the ultimate strength of the concrete and increase its expansion under water and shrinkage under drying conditions.

### Densities of concrete samples

**Figure 5:**
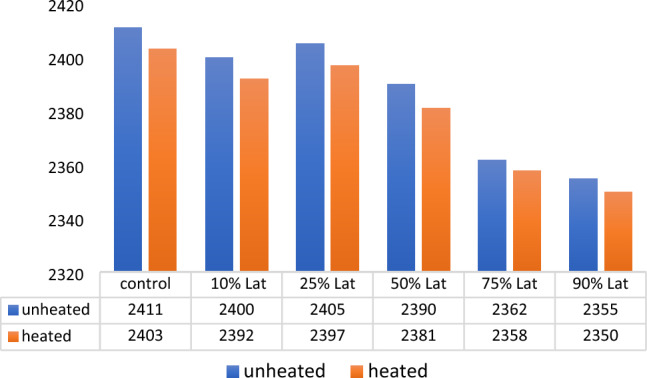
Densities of hardened concrete samples at 28 days.

Density is an important property of concrete that determines its mass per unit volume. the density of concrete can vary depending on the composition of its constituents and the specific mix design. Understanding the density properties of concrete is essential for various aspects, including structural design, load calculations, and material specifications. It allows engineers and contractors to assess the weight, mass, and performance characteristics of concrete in different applications. The densities of hardened concrete cube samples shown in Fig. 5 were found to range from 2345 to 2411 kg/m^3^ which indicate concrete for structural and load bearing purposes and showed that the density decreases with increase in lateritic soil percentages in the concrete mix. The declining density of laterite concrete samples as lateritic soil content increases is mainly because of factors like reduced particle density, higher porosity, increased water absorption, variable particle size distribution, aeration, air voids, and chemical interactions. Overall, the unheated specimens possessed higher density properties than the heated samples though some of the results for unheated appear greater than normal weight concrete. This may be due to excess water from curing tank, since the samples were only surface dried before weighing^69^.

### Compressive strength development

**Figure 6:**
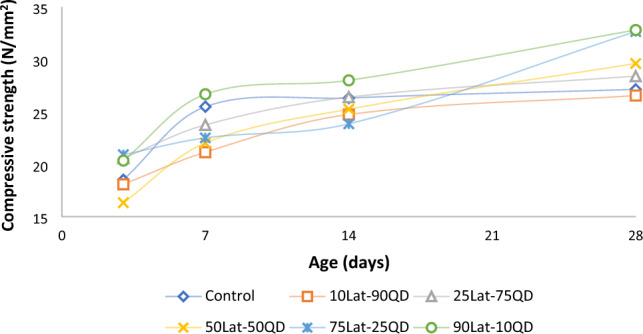
Development of compressive strength of concrete (unheated).

Compressive strength is a vital property of concrete that measures its ability to withstand compressive forces and is an important parameter for structural design and evaluation. The compressive strength development properties of concrete is essential for designing and assessing the durability performance of concrete structures. It helps in determining the appropriate curing conditions, evaluating the early-age strength gain, and ensuring the long-term durability and structural integrity of concrete elements^70^. The development of compressive strengths for various unheated concrete mixes cured at 3, 7, 14, and 28 days respectively are presented in Fig. 6. The results show that compressive strengths increased with increase in the laterite content from at 3 days to 28 days curing with 18.51–26.55 N/mm^2^, 18.1–26.55 N/mm^2^, 20.20–28.4 N/mm^2^, 16.33–29.61 N/mm^2^, 20.91–32.65 N/mm^2^, and 20.34–32.80 N/mm^2^ for control, 10% lat., 25% lat., 50% lat., 75% lat. and 90% lat. respectively. The results were generally higher than the control concrete, except for the mix with 10% laterite, which was marginally less than the control. These results appear to contradict earlier findings by Ukpata et al.^3^, where compressive strengths were found to increase with decrease in laterite contents. This may be attributed to the difference in the quality of lateritic sand used in the study.

### Effects of elevated temperature on compressive strengths

**Figure 7:**
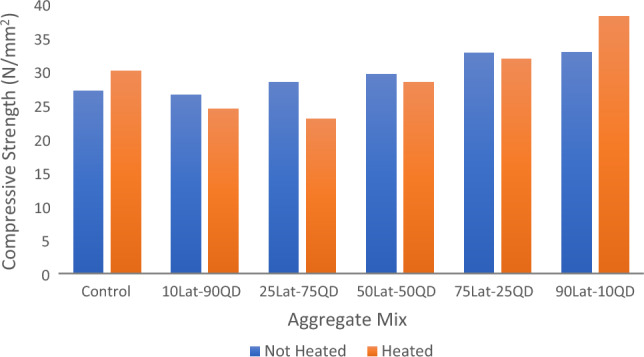
Effect of heating on 28-day compressive strength.

The effects of elevated temperature on the compressive strength of concrete are significant and can have detrimental consequences on the structural performance of concrete. The effects of elevated temperature on the compressive strengths of concrete is vital for assessing the fire resistance and thermal performance of concrete structures. Proper fire protection measures and design considerations are necessary to mitigate the adverse effects and ensure the structural integrity of concrete under high-temperature conditions. The comparison between the compressive strengths of heated and unheated concrete samples cured for 28 days is shown in Fig. 7. The result shows that the higher the laterite content the higher the compressive strength when heated except for 10% laterite. It also shows that compressive strengths of unheated samples are higher than the heated ones for various percentages of laterite ranging from 10 to 90% replacement except for 90% where the heated sample has a higher compressive strength than the unheated corresponding to that of the control. These results appear to contradict earlier findings by Surya et al.^71^, where compressive strengths were found to increase upon heating less than 500 °C temperature.

### Effects of elevated temperature on flexural strengths

**Figure 8:**
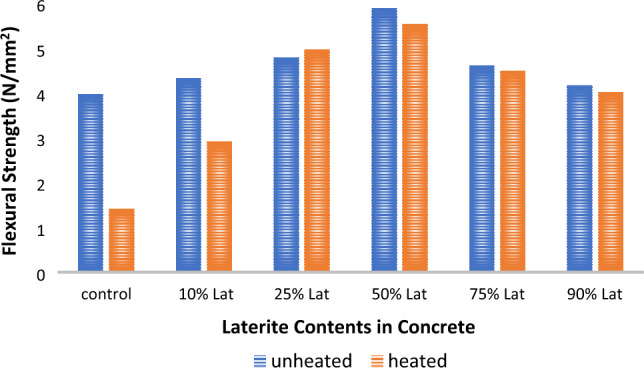
Effect of heating on 28-day flexural strength.

Elevated temperatures can have significant effects on the flexural strength of concrete, which is a fundamental property for assessing the structural performance and resistance to bending. The results presented in Fig. 8 illustrate that the flexural strength of 28-day-cured concrete increases with a higher laterite content. The maximum strength responses, 5.89 N/mm^2^ for unheated specimens and 5.54 N/mm^2^ for heated specimens, are achieved at a 50% laterite addition to the mix. However, exposure to elevated temperatures leads to a decrease in flexural strength, and this reduction is more significant when the mix contains lower levels of lateritic soil from 0 to 10%. Interestingly, the flexural strength of the concrete heated with 25% lateritic soil content surpasses that of the unheated concrete sample. As the lateritic soil content exceeds 25%, the differences between the heated and unheated samples become smaller^72^.

### Sorptivity of heated and unheated samples

**Figure 9:**
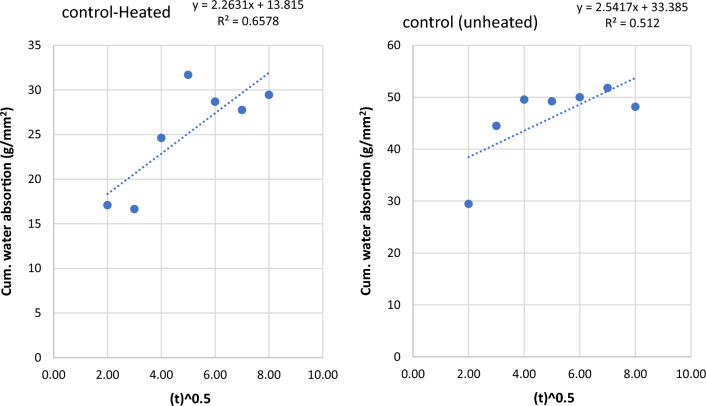
Graph of cumulative water absorption versus square root of time (control heated and unheated).

**Figure 10:**
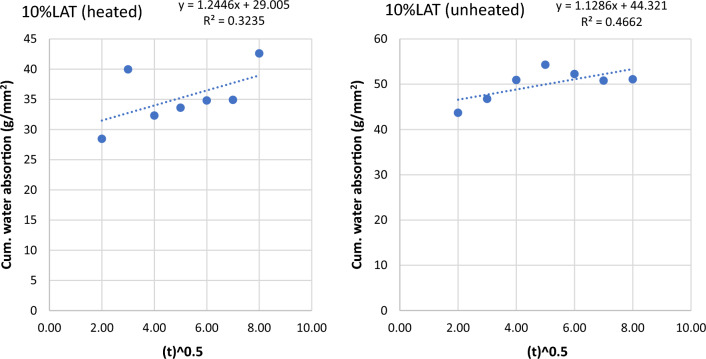
Graph of cumulative water absorption versus square root of time (10% laterite heated and unheated).

**Figure 11:**
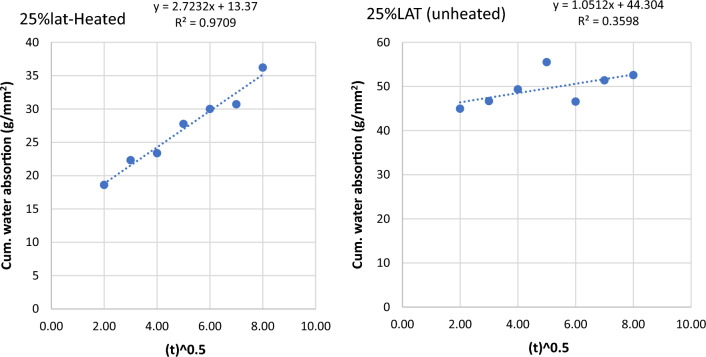
Graph of cumulative water absorption versus square root of time (25% laterite heated and unheated).

**Figure 12:**
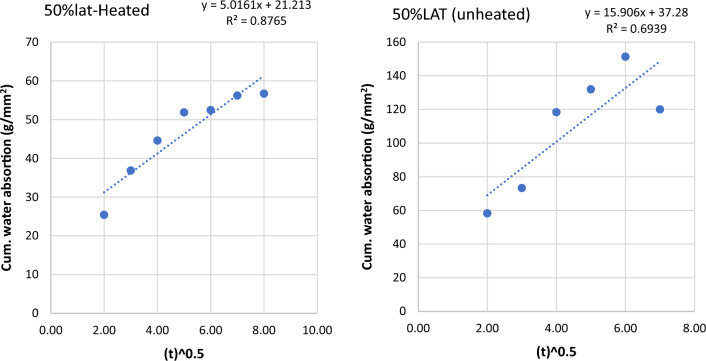
Graph of cumulative water absorption versus square root of time (50% laterite heated and unheated).

**Figure 13:**
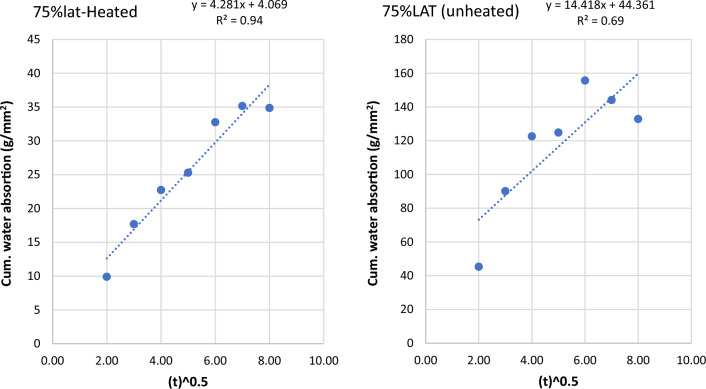
Graph of cumulative water absorption versus square root of time (75% laterite heated and unheated).

**Figure 14:**
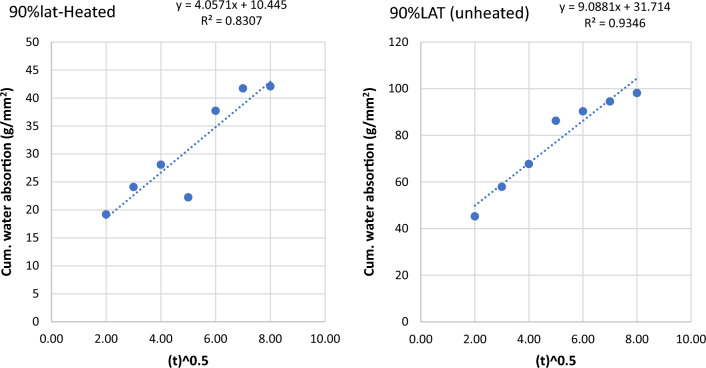
Graph of cumulative water absorption versus square root of time (90% laterite heated and unheated).

The sorptivity of heated and unheated laterized concrete samples refers to the rate at which water is absorbed by the concrete after it has been subjected to heating. Sorptivity is an important property to evaluate the permeability and moisture transport characteristics of concrete. The sorptivity of heated and unheated laterized concrete samples is important for assessing their durability and moisture-related performance. It helps in designing appropriate waterproofing and moisture protection measures for concrete structures to prevent water ingress and potential damage. The graphs of cumulative water absorption were plotted against the square root of time (in minutes) to determine water sorptivities for the concrete samples. The graphs are shown below from Figs. 9, 10, 11, 12, 13, 14.

**Figure 15:**
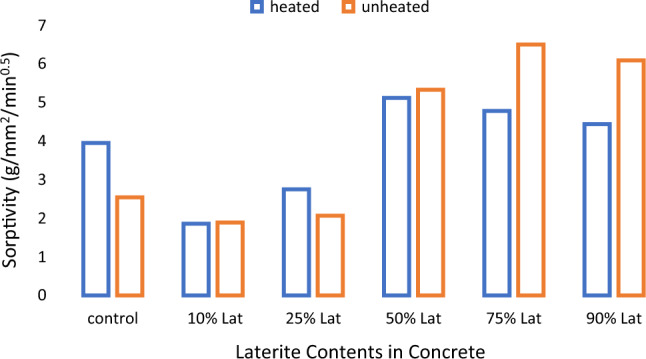
Effects of heating on sorptivity × 10^–5^.

The effects of heating to elevated temperatures on sorptivities of concrete samples is presented in Fig. 15. Heating to elevated temperature led to increased sorptivities for control samples and samples with low laterite contents up to 25%. Generally, laterite contents from 50 to 90% led to reduced sorptivity in the heated samples. This suggests some refinement of the microstructures leading to decreased ingress of water into the heated samples. This may explain the improvements in some mechanical properties shown earlier.

### Microstructure of concrete samples

The microstructure of concrete refers to the arrangement, composition, and characteristics of its constituent materials at the microscopic level. Understanding the microstructure is essential for assessing the properties and performance of concrete. The microstructure of concrete is crucial for optimizing its design, assessing its performance, and developing strategies for improving its properties and durability. Techniques such as microscopy, scanning electron microscopy (SEM), and other imaging methods are commonly used to study the microstructure of concrete samples. Microstructural studies were also employed to understand the behaviors of the various samples concerning mechanical and water transport properties. Hence, scanning electron microscopy (SEM), coupled with energy dispersive x-ray (EDX) spectroscopy were used to study the microstructure of concrete samples. These were supplemented by Fourier Transform Infra-red (FTIR) spectroscopy^73^.

#### SEM–EDX results

**Figure 16:**
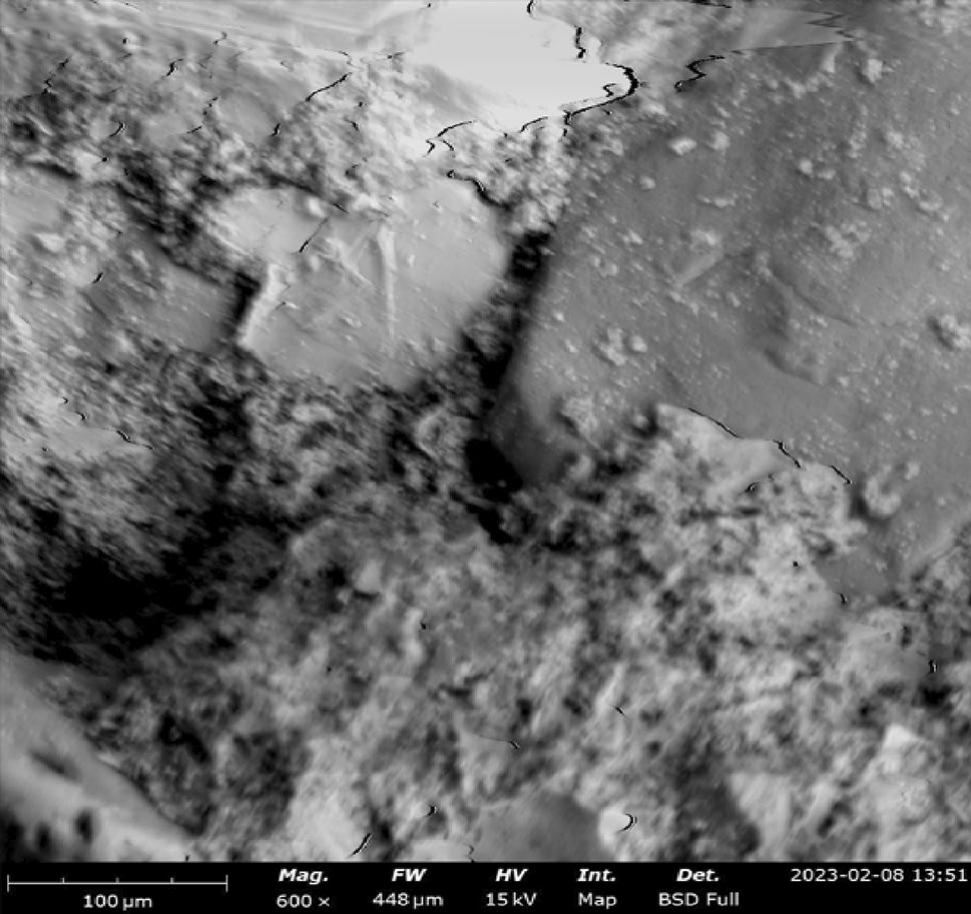
SEM image of control sample (not heated), after mechanical testing.

**Figure 17:**
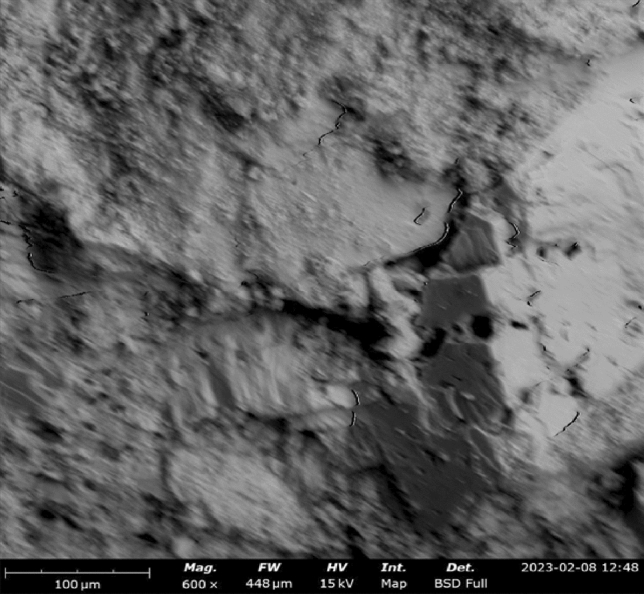
SEM image of concrete sample with 90%-laterite/10%-quarry dust as fine aggregate (not heated).

**Figure 18:**
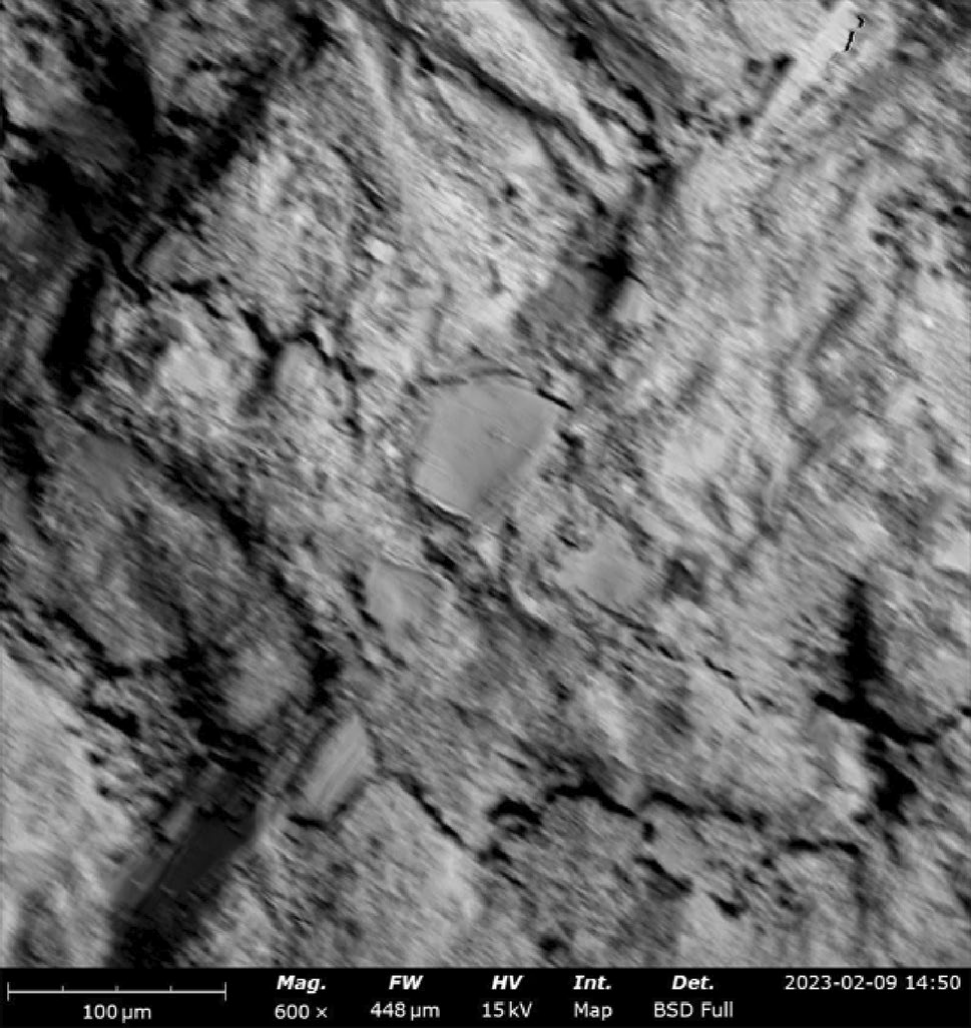
SEM image of concrete sample with 90% laterite/10% quarry dust as fine aggregate (heated).

SEM–EDX (Scanning Electron Microscopy with Energy-Dispersive X-ray Spectroscopy) is a powerful technique used to analyze the microstructure and chemical composition of materials, including concrete. By combining SEM imaging with EDX analysis, detailed information about the elemental composition and distribution within concrete samples can be obtained. Figures 16, 17, 18 show the SEM images of concrete samples selected for the control, concrete sample with 90%-laterite/10%-quarry dust as fine aggregate (not heated), and concrete sample with 90%-laterite/10%-quarry dust as fine aggregate (heated). The control sample shows hydrated and unhydrated phases, with some cracks. A comparison between non heated and heated samples can be seen in Figs. 17, 18 respectively. Despite some cracks, the heating had accelerated hydration activities, leading to a more compact microstructure, which in turn led to increased compressive strength shown earlier.

**Figure 19:**
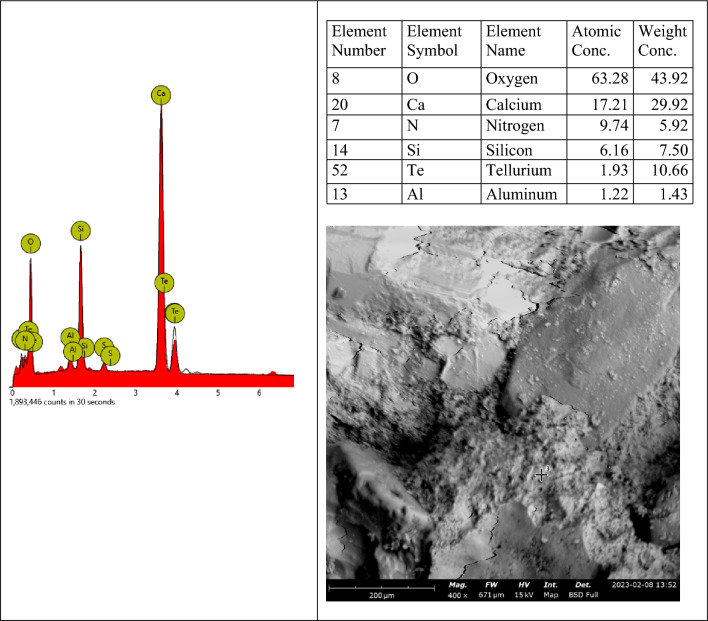
SEM–EDX elemental spot 1 analysis of control sample.

**Figure 20:**
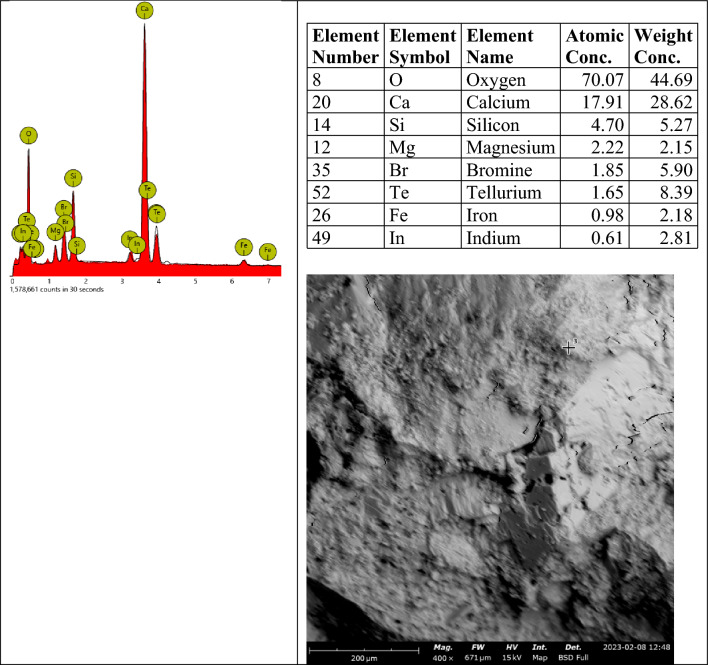
SEM–EDX elemental spot 1 analysis of 90% laterite/10% quarry dust as fine aggregate (not heated).

**Figure 21:**
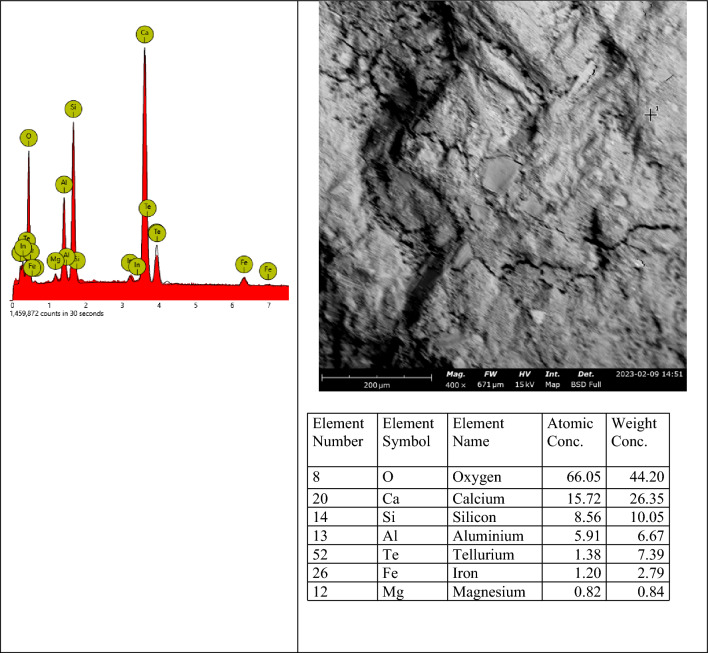
SEM–EDX elemental spot 1 analysis of 90% laterite/10% quarry dust as fine aggregate (heated).

The elemental EDX spot analysis conducted on the SEM samples has revealed the elements associated with the analyzed points as shown in the EDX spectra in Fig. 19. The EDX spectrum shows predominantly silicon, oxygen and calcium, including other elements, suggesting hydrated calcium-silicate-hydrate (C-S-H), which agrees with the point indicated in the SEM image. The SEM images in Figs. 20 and 21 show different microstructures from the control sample due to the high volume of laterite. Nevertheless, the presence of C-S-H appears to be the captured spot. The presence of laterite intermixed phase is confirmed by the presence of iron element.

#### FTIR results

**Figure 22:**
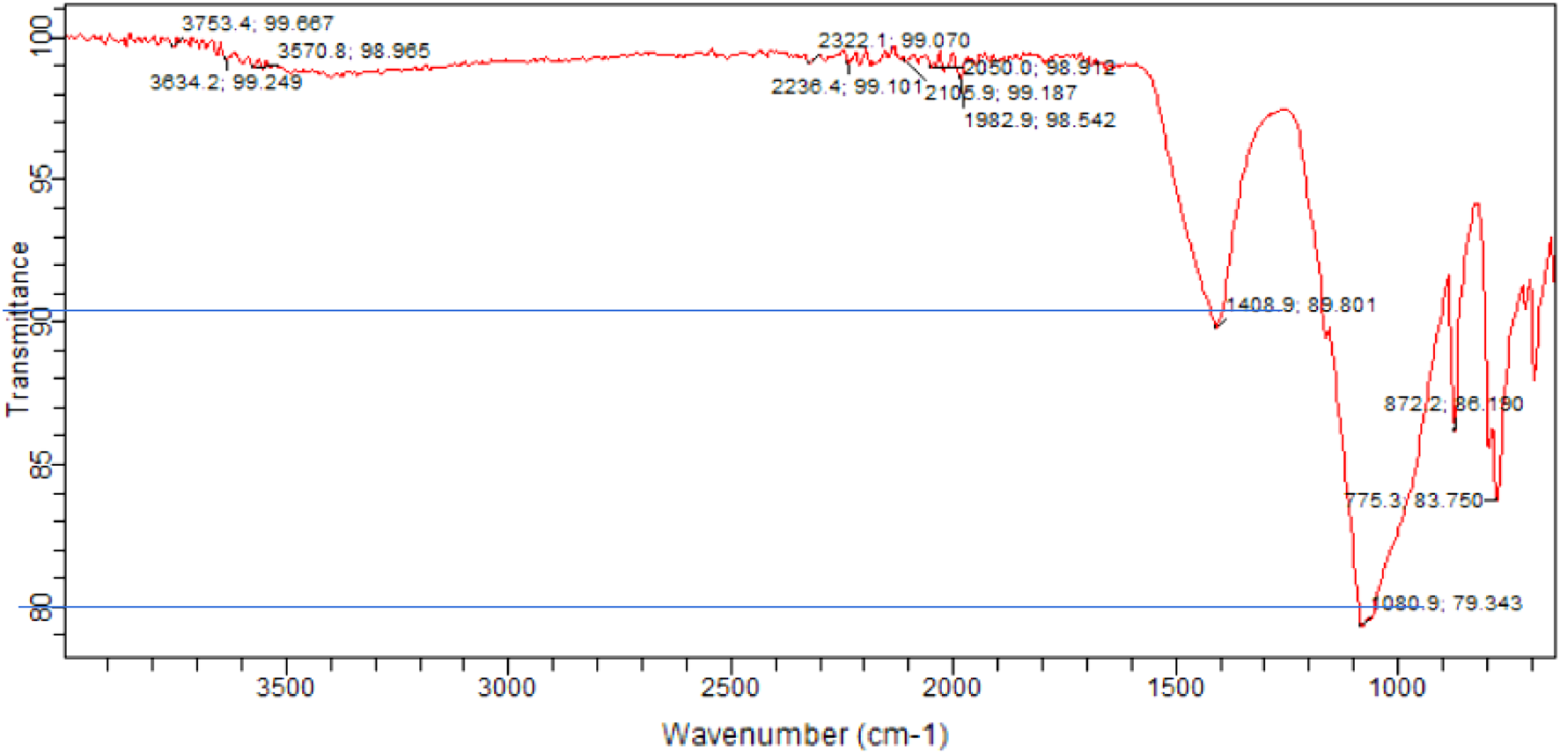
FTIR spectra of control concrete with sand as fine aggregate.

**Figure 23:**
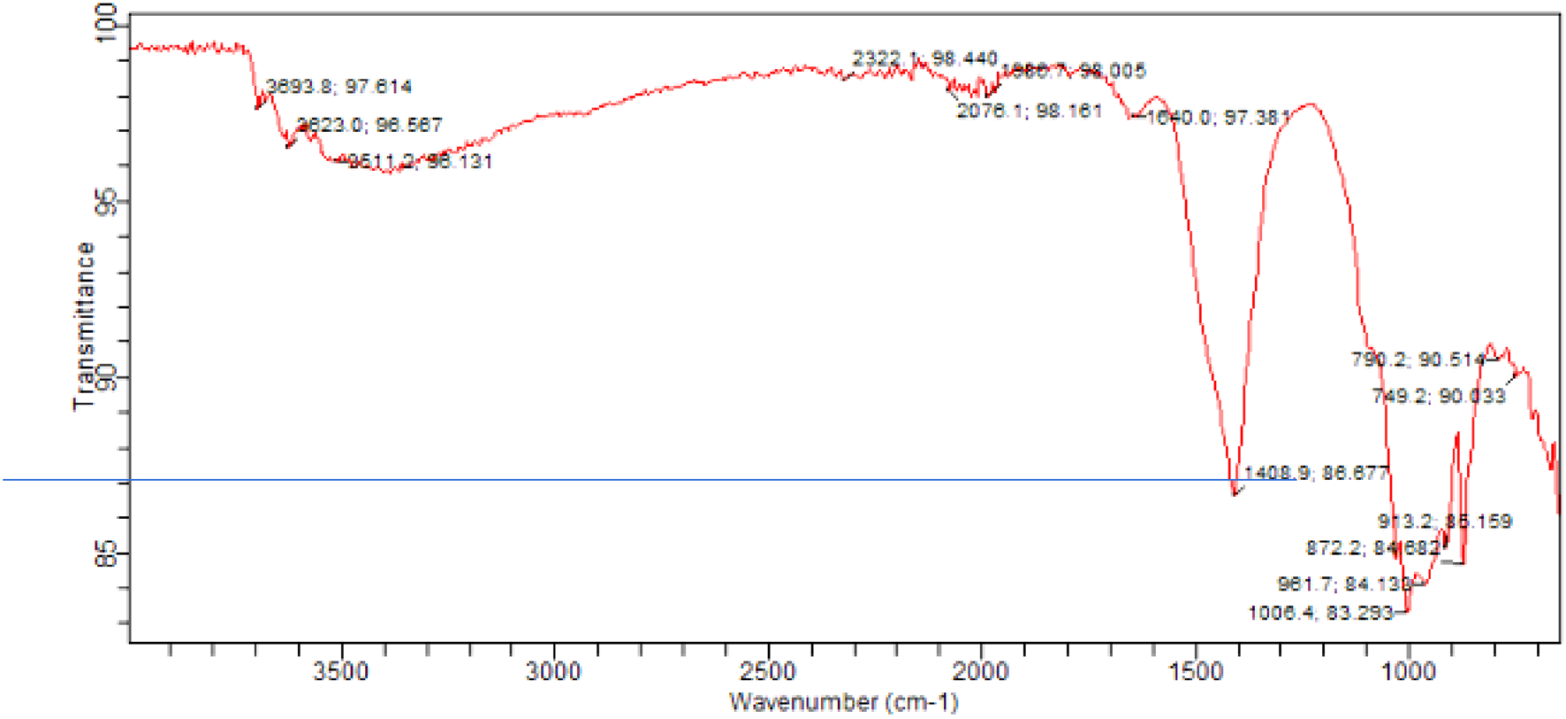
FTIR spectra of concrete with 90% laterite/10% quarry dust as fine aggregate (not heated).

**Figure 24:**
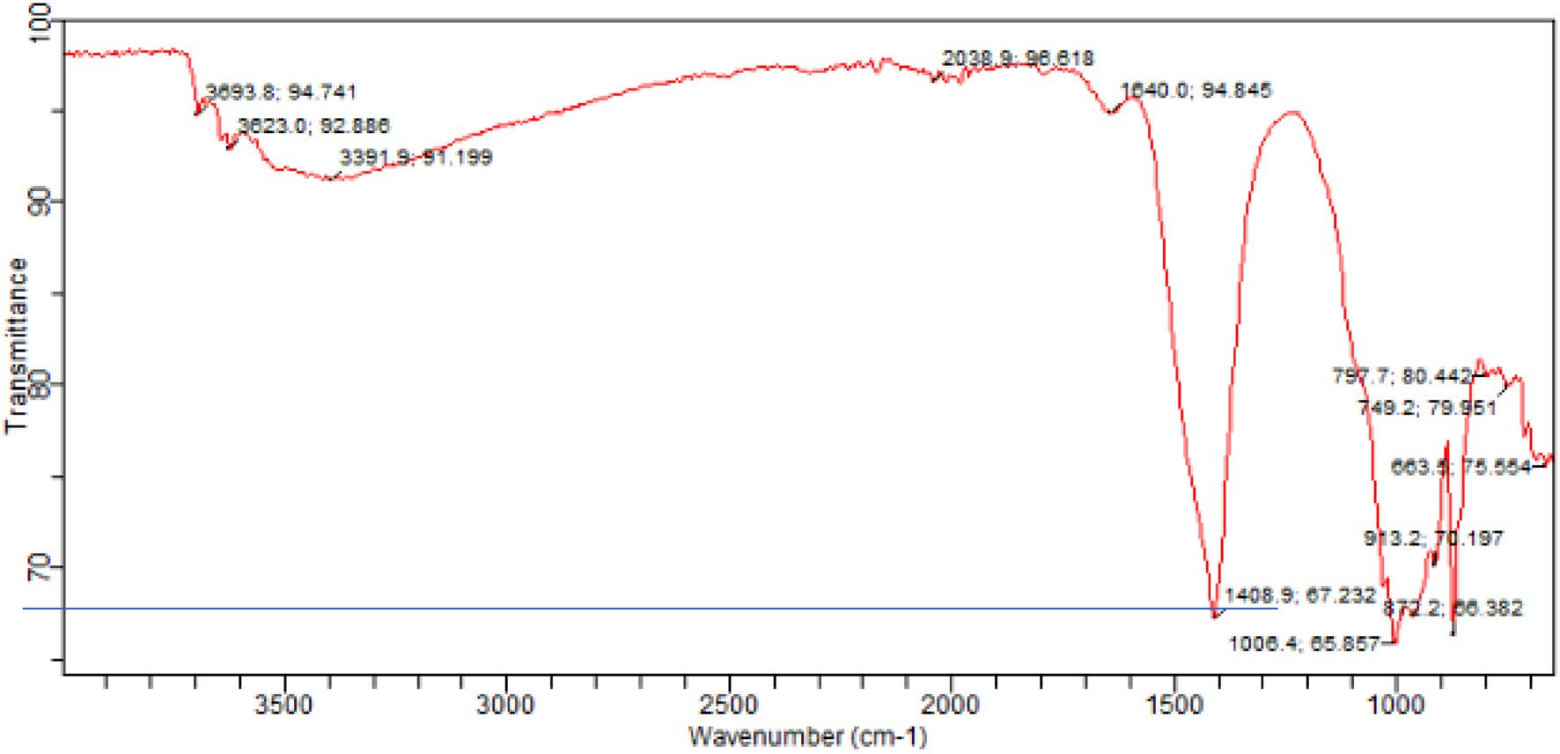
FTIR spectra of concrete with 90% laterite/10% quarry dust as fine aggregate (heated).

FTIR (Fourier Transform Infrared) spectroscopy is a technique used to analyze the molecular composition and chemical bonds present in a material. It can also be applied to study concrete and provide valuable information about its composition and chemical characteristics. Fourier Transform Infrared (FTIR) spectra for corresponding samples as for SEM are presented in Figs. 22, 23, 24. The major reflections correspond to Si–O (Afwillite, C-S-H), C-O (calcium carbonate), S–O (ettringite), and O–H (portlandite) bonds respectively. The reflections are generally less intense in the control sample (Fig. 22) than the samples with high laterite load (Figs. 23, 24). This is however different for ettringite which reflection was more intense in the control sample than laterite dominated samples. This suggests the consumption or inhibition of monosulfates in Portland cements due to pozzolanic reactions. For the laterite dominated samples, the heated samples Fig. 24, show more intense reflection for calcium carbonate due to heating. The reflection corresponding to C-S-H appear more intense in the heated samples (Fig. 24), than unheated samples (Fig. 23), which tend to support the increased strength observed^74^.

## Conclusion

This study has investigated the structural properties of concrete containing laterite fine aggregate and the effects of heating to elevated temperature on the residual structural, microstructural and durability properties of concrete samples. It can be seen from the results of this study that the combination of laterite and quarry dust to replace the conventional river sand in the production of concrete for the construction industry in Nigeria and other tropical countries of the world results in structures with reliable structural characteristics and should be encouraged where there is comparative cost advantage. The physical property tests on the aggregates indicated a specific gravity result of 2.6, 2.6, 2.2 and 2.9 for sand, quarry dust, laterite and crushed granite respectively. The aggregate impact value test showed that the coarse aggregate used for the study was exceptionally strong having a value of 6.7% which is less than 10% according to BS EN 1097-2:2010.

The compressive and flexural strengths of concrete using lateritic sand and quarry dust were measured in the laboratory. Compressive strength of the concrete was found to increase significantly when heated up to 250 °C and also increased with age as it is with conventional concrete. It was discovered that the compressive strength of the concrete at 28days varied with respect to the replacement percentages ranging from 26.55 to 32.8 N/mm^2^ for room temperature, and 23–38.13 N/mm^2^ for elevated temperatures; flexural strength ranged from 3.88 to 5.98 N/mm^2^ and 1.43–5.54 N/mm^2^ for unheated and heated specimens respectively. The above strength properties were found to compared closely with normal concrete, while the proportion of 90% laterite to 10% quarry dust produced higher compressive strength values, 50% laterite to 50% quarry dust however produced better flexural strength.

### Contribution to knowledge

This study has added important contributions to knowledge in numerous ways. This is the first known work to conduct the Fourier Transform Infrared Spectroscopy (FTIR) analysis which provides the chemical properties, and the Scanning Electron Microscopy (SEM), which gives the microstructure of the hardened concrete aggregate constituents with this replacement percentages within the geographical region investigated. The work will trigger further studies into the use of lateritic sand and quarry dust as fine aggregate in the production of structural concrete. This work has also directed interest in new areas for recycling of quarry dusts which solves environmental waste problems.

### Recommendations for further studies

Further research should be conducted to provide more design data for use. Therefore, the following area is recommended for further studies:

i. The effects of elevated temperatures on the residual elastic modulus properties of concrete produced using laterite and quarry dust as fine aggregate
ii. Addition of supplementary cementitious materials obtained from waste derivatives in the development of laterized concrete
iii. Application of artificial intelligence modelling for the optimization of the effects of elevated temperature on laterized concrete.

## Data Availability

All data generated or analyzed during this study are included in this published article.

## Abbreviations

Lat: Limestone
QD: Quarry dust
W/C: Water cement ratio
C-S-H: Calcium silicate hydrate
SEM: Scanning electron microscopy
EDX: Energy dispersive x-ray
FTIR: Fourier transform infrared spectroscopy
XRF: X-ray fluorescence
